# Organisational Strategies for Women Nurses to Advance in Healthcare Leadership: A Systematic Review

**DOI:** 10.1155/2023/2678916

**Published:** 2023-12-07

**Authors:** Mihirika Surangi de Silva Pincha Baduge, Mariam Mousa, Belinda Garth, Leanne Boyd, Helena J. Teede

**Affiliations:** ^1^Monash University, Melbourne, Australia; ^2^Eastern Health, Melbourne, Australia

## Abstract

**Aim:**

We aimed to undertake a systematic review focusing on organisational strategies that specifically advance women nurses in healthcare leadership.

**Background:**

Despite nursing being the largest health workforce and being dominated by women, they face significant barriers in career progression and have limited leadership opportunities, with a need to move from a focus on individuals to organisational level change.

**Methods:**

Methods for our overarching systematic review are published and follow the Preferred Reporting Items for Systematic Reviews and Meta-Analyses (PRISMA) checklist. Databases searched include MEDLINE via OVID; MEDLINE in-process and other nonindexed citations via OVID; PsycINFO; and Scopus. Any study describing a strategy that was not implemented for nurses and all non-peer-reviewed studies were excluded. Included studies were appraised using CASP checklists. A thematic analysis approach was employed to facilitate the systematic generation of themes.

**Results:**

Findings were reported under two main themes: leadership barriers and enablers and organisational strategies for advancing women nurses to leadership. The latter included: mentorship, leadership training, career planning and coaching, opportunities for networking, sponsorship, and targeted recruitment processes for increasing gender diversity in leadership roles.

**Conclusion:**

This review synthesises organisational-level strategies that advance women nurses in healthcare leadership. Barriers and individual-level strategies for advancing women nurses in healthcare leadership have been extensively studied in the current literature. Systems and organisational strategies are less studied yet show that they can advance women in nursing into healthcare leadership. *Implications for Nursing Management*. This paper suggests that optimising women nurses' leadership attainment needs to shift focus from individual strategies to systemic level and organisational strategies and use tailored evidenced-based approaches.

## 1. Background

Nursing constitutes the largest healthcare workforce, and despite being majority women, there is an underrepresentation of women nurses in healthcare leadership [1, 2]. The global nursing workforce includes nearly 27 million nurses and midwives, or 50% of health professionals [3]. Importantly, however, there is very little representation of nurses in leadership positions. United States shows that nurses represent only 2–6% of the healthcare boards [4]. Yet nurses in leadership have been shown to offer benefits and unique perspectives in “strategic planning, critical thinking, communication, quality and process improvement, human resources, finance, and complex problem solving” [5]. Extensive evidence demonstrates the impactful role of nurse leaders in various healthcare outcomes for patients, organisations, the nursing profession, and the wider community [6–8]. Nurses are central to achieving the Sustainable Development Goals of achieving universal health coverage for all [2, 9]. Having gender equity in nursing leadership offers benefits by breaking down gender stereotypes and demonstrating the capacity of women to lead in healthcare [1]. It can benefit organisations by increasing diversity, bringing different perspectives to the decision-making process, and promoting greater gender equality in society at large [6, 10]. This highlights the need to explore the constraints present in the healthcare sector that hinder women nurses from participating in healthcare leadership.

Leadership is often traditionally viewed as masculine traits and values such as “agency, assertiveness, and decisiveness.” The medical dominant model of healthcare leadership also disadvantages women nurses [1, 10], impacting “perceived credibility, capability, and capacity” for women nurses in leadership roles [11]. The positions of nurse leaders are typically seen as distinct from clinical roles, and leadership and management terms are used interchangeably [12]. The concept of nursing leadership is now shifting beyond the traditional authoritative style to a more collaborative and people-centred approach that encourages and empowers peers and drives change. Nursing leadership involves a more collaborative approach at different levels rather than being in silos within a physically demarcated unit. Nursing leadership is not defined by a physical landmark as a ward or unit and is not limited to the head nurse, nurse-in-charge, or director of nursing titles; it entails leadership at all levels, including clinical leadership, middle-level clinical managers, nurse executives, academic leaders, the public sector, and policy-level leaders, allowing nurses to practice to the full extent of their education and training [6, 9]. In this study context, nursing leadership entails fostering a culture of excellence that supports the best possible patient outcomes. It is about driving change, fostering collaboration, and empowering nursing professionals to thrive in a rapidly evolving healthcare landscape. Furthermore, nursing leadership is thought to be a key determinant of job satisfaction among nurses [13].

The nursing profession itself is gendered, impacting perceived value, recognition, and leadership identity. As a traditionally perceived caring role, nursing is often stereotyped as feminine work with fewer men choosing a career in nursing and with the profession itself being gendered [1]. The intersection of nursing and women gender contributes to unconscious gender bias, both leading to inequitable broader leadership opportunities in healthcare for women nurses [1, 2, 14]. In this context, the “*glass escalator*” also sees men nurses ascend the leadership ladder faster than their women counterparts [1]. An example of this can be seen in research from developed countries such as Australia where, despite making up only 9% of the nursing workforce, men represent 14% of mid-level managerial roles (e.g., nurse supervisor, nurse unit manager, or charge nurse) and 12% of policy roles [15]. The nursing workforce is also often ethnically diverse, bringing additional intersectional barriers to leadership attainment [1].

While strategies for women's career advancement have been studied, they often focus on individual fixes rather than addressing systemic organisational issues as highlighted in the Lancet special issue on *Advancing women in science*, *medicine*, *and global health* [10, 11, 16, 17]. Organisational culture is often created by men for traditional men-gendered roles, and women are expected to function and flourish on their own in a system that is not well suited to advancing women's careers [10, 18]. Organisational reform is increasingly understood to be essential to tackling gender-related systemic issues [16, 18]. Strategies for nurses to attain leadership positions are studied and reported on without a specific gender lens [19, 20] and are studied in other healthcare disciplines, including medical disciplines and the broader healthcare sector [18, 21]. However, unique barriers and facilitators affecting women nurses and organisational-level strategies to improve women nurses' leadership are understudied. Therefore, knowing the organisational strategies and the systemic-level constraints present in the nursing profession and within an organisation will help identify remedies and resolutions to targeted problems. Exploring the organisational strategies and systemic-level barriers will help identify and guide enablers for change. These priorities were also established with the National Health and Medical Research Council (NHMRC)C project partners including large health services and nursing organisations. Stakeholders highlighted that creating conducive culture organisations can optimise empowerment of women nurses to achieve their full potential and lead in healthcare. This systematic review explores the evidence for organisational-level strategies that advance women nurses to higher leadership.

In this context, we build on a recent systematic review [21], focusing on organisational-level strategies that advance women nurses in healthcare leadership. We aim to identify evidence-based organisational strategies relevant to the nursing profession through a systematic review and narrative synthesis. We also aimed to capture documented barriers and enablers to women nurses' advancement in healthcare leadership.

What are the barriers and facilitators that are shaping the implementation of strategies?

What are the organisational-level strategies that advance women nurses in healthcare leadership?

## 2. Methods

Methods for our overarching systematic review are published and follow the Preferred Reporting Items for Systematic Reviews and Meta-Analyses (PRISMA) checklist (Table S3 and Table S4) [22]. Databases searched include MEDLINE via OVID; MEDLINE in-process and other nonindexed citations via OVID; PsycINFO; and Scopus. Terms included leadership, OR career mobility, OR career progression, OR career advancement, AND academia, OR health services, AND female, OR women. Here, articles were included only if they (1) examine a strategy implemented for the healthcare workforce, which includes women nurses; (2) describe organisational strategies and practices; (3) report any strategy designed for women healthcare professionals, that included nurses, for advancing leadership; (4) report on a measurable outcome; and (5) were published in English between January 2000 and March 2021. We excluded all non-peer-reviewed studies and any study describing an intervention that was not implemented for nurses and not focused on leadership. Based on CASP checklists, the included studies were graded 2, 1, or 0 based on methodological rigor and rated high, moderate, and low quality (MM and MP). No studies were excluded based on the quality. Risk of bias assessment at the study level is provided in Supplementary Table 2.

### 2.1. Data Extraction and Analysis

Due to the heterogeneity of data, meta-analysis was not possible and a narrative synthesis was undertaken. Narrative synthesis summarises and explains findings by using words and text synthesis from multiple studies to understand “the effects of interventions and/or the factors shaping the implementation of interventions” [23]. The stages outlined in the Guidance on the Conduct of Narrative Synthesis in Systematic Reviews was followed for the preliminary analysis [24]. Key study data were extracted and tabulated, including author, year of publication, country, journal, methodology, population characteristics, and methods of data collection and analysis (see Supplementary Table 1). This was followed by data synthesis and analysis.

The initial analysis was performed by one of the authors (MP) and derived codes were discussed among coauthors. Initial codes were inductively identified using a data-driven process where data are not forced into a preexisting framework or researcher's analytical presumptions [25]. Types of interventions were colour coded by using highlights. Organisational strategies were extracted and Table 1 created to vote count reported organisational strategies. A code sheet was developed in Microsoft Excel. The deductive approach was used in subsequent papers and was open to capture new codes that emerged from the data. Similar codes were clustered into groups to identify potential themes. For this substudy, only studies related to nursing were extracted. Any additional codes related to nursing were extracted by MP, and a dedicated narrative synthesis was completed and confirmed with other authors (MM, BG, and HT).

## 3. Results

The overarching systematic review by Mousa et al. [21] identified 5517 articles for screening, with 4757 titles and abstracts screened after removing duplicates, 567 full reviews, and 91 selected studies. This substudy included six studies focused on nursing. Six studies met the eligibility criteria and were included in the review. Data were extracted on barriers and facilitators to leadership and potentially effective organisational-level strategies, and the data were reported under these themes (Figure S2). Supplementary Figure 1 shows the PRISMA flowchart for retrieved, excluded, and included papers. Across all professions, six studies met inclusion criteria in nursing leadership.  Table 2 shows key results.

### 3.1. Description of Included Studies

#### 3.1.1. Study Characteristics

The focus of the included studies was nurses (*n* = 3) or healthcare with nursing involvement (*n* = 3). Included studies were qualitative (*n* = 3) and mix-method (*n* = 3) studies. No quantitative studies were found. Two out of six included studies were conducted in the USA, three were from the UK, Australia, and Canada respectively, and one was an international study where participants represented 68 countries. Of the three nursing studies, one focused on early career academic nurses' barriers to advancement, the second focused on clinical mid-career nurses' leadership training, and the third investigated mentors' insight into the mentorship program. The three studies that were healthcare with nursing involvement were conducted on advancing women in global healthcare leadership, leadership development, and women Chief Executive Officer's (CEO) career inflection points.

#### 3.1.2. Participant Characteristics

Sample sizes ranged from 12 to 405, totalling 620 participants across all studies with 90% (*n* = 557) women and 10 (*n* = 63) men. Participants included mental health nurses (*n* = 24), Chief Executive Officers (CEOs) (*n* = 20), early career nursing academics (*n* = 23), healthcare workers and healthcare leaders from 68 countries (*n* = 405), graduates from faculty leadership development program (*n* = 136), and mid-career nurses (*n* = 12).

### 3.2. Leadership Barriers and Enablers

#### 3.2.1. Leadership Barriers

Barriers for women nurses to reach leadership roles were reported among early career academics, senior clinicians, and in global nursing across all levels [27, 28, 31]. The reported barriers were categorised into systemic- and individual-level barriers.

“Women face unique barriers to advancing to positions of global health leadership compared to men.” [28]

Senior clinicians experienced a glass ceiling for higher leadership roles and also noted dependence on the culture and organisational leadership [31]. Furthermore, intersections within nursing and normalisation of men leadership styles were also reported barriers for middle and lower-income countries [28]. Halcomb et al. [27] reported dearth of available leadership training and mentoring programs focusing on nurse academics. Additionally, senior women clinicians felt frustrated due to the inability to implement change, inability to take issues forward, less clarity over career pathway for management positions, and unavailability of internal leadership training [31]. In terms of global nursing leadership, lack of mentorship, gender bias, lack of women mentors, feeling less confident, and lack of assertiveness were reported among early, mid-, and senior nurses, regardless of country income level [28]. In addition, in lower- and middle-income countries, nurses experienced a lack of leadership opportunities and training, less funding and networking opportunities, higher workload, and challenges with travel requirements and safety concerns [28].

In terms of individual-level barriers, even though early career nurse academics held clinical leadership roles, they did not view themselves as leaders in their new academic roles [27]. Additionally, early career nurse academics were perceived as less confident and less autonomous in their transitional role [27]. Furthermore, lack of assertiveness, poor understanding into teaching-learning practices, and role and expectations as academics were also reported as barriers [27]. Women nurses were also challenged with work-life balance at an individual level.

#### 3.2.2. Leadership Enablers

Three studies reported leadership enablers [27, 29, 31]. Providing leadership opportunities at the early career stage facilitated leadership advancement [27, 29]. Having broad experiences in a variety of settings was reported as a facilitator [29]. Management training and formal training for mentors were identified as lead facilitators for senior clinical women nurses [31]. Having a professional graduate degree/leadership training/executive coaching, mentorship, sponsorship, networking, family support, career planning, and taking risks were individual factors that are positively associated with healthcare leadership advancement in women nurses [29].

### 3.3. Organisational Strategies for Advancing Women Nurses to Leadership

“We have largely focused on individual-level interventions to overcome (women's barriers to advancement) rather than looking at how we can change organizations, systems, and policies to better overcome these barriers. We need systemic interventions if we are going to make an impact.” [28]

Systemic-level strategies rather than individual-level strategies were highlighted as key for changing organisations, systems, and policies to make an impact and overcome barriers to women's advancement to leadership [28]. Systemic strategies such as mentorship, leadership development programs, and networking opportunities were found to be potentially effective across most levels and settings [27–31].

Table 1 shows the core interventions captured in this systematic review, across mentorship, leadership development and training, sponsorship, networking opportunities, and interventions to create opportunities. Mentorship and leadership training in particular were identified across numerous studies.

#### 3.3.1. Mentorship

Out of six included studies, four reported mentorships as a potentially effective organisational-level strategy for advancing women in healthcare leadership [27–29, 31]. Woolnough et. al. [31] found that mentors also benefited from an organisational perspective with “increased understanding of the mentoring role, increased awareness of career barrier for female mental health nurses, improved ground-level insight in relation to nursing staff and the patient they cared for, improved professional reputation, increased networks, new insights into organisational issues, personal enjoyment and fulfilment, and desire to implement organisational change.” Mentoring offered during the early career stage profoundly impacted executives' career trajectories and provided incentives for mentorships and for selectively recruiting women nurses into training programs by providing networking opportunities to women nurse leaders [28, 29].

#### 3.3.2. Leadership Training

Four studies reported leadership training and educational strategies as potentially effective organisational-level strategies for advancing women in healthcare leadership [27–30]. Leadership training designed for women helped address women nurses' perceived lack of assertiveness [28]. Additionally, Tsoh et. al. [30] noted that leadership training went beyond covering leadership models and focused on models such as “Coro Northern California (Coro) Experiential Training,” highlighting the individual, interpersonal, and institutional level impact of leadership.

Additionally, career assistance, orientation programs, shared experiences, and stories of successful senior nurse academics were reported to be supportive organisational-level strategies for early career nurse academics [27]. Donner and Wheeler [26] argued that to retain mid-career nurses, organisational-level strategies are required focusing on their personal and professional development such as providing opportunities for their career planning and structured career coaching. Executive training programs were found to be potentially effective leadership development intervention for senior clinicians [29]. The necessity for available leadership training via healthcare organisations is highlighted in some studies [27, 28].

## 4. Discussion

In terms of organisational strategies for advancing women nurses in healthcare leadership, mentorship, leadership training, leadership opportunities, sponsorship, career planning/coaching, providing networking opportunities, targeted recruitment to improve women's representation in leadership roles, and orientation to leadership roles were found to be potentially effective [26–31].

Mentorship is an established leadership enabler [1, 10, 21, 32]. Mentoring is an active process that occurs between two people: mentee and mentor. The aim of the mentee is to acquire new knowledge, skills, and opportunities to flourish in a new professional environment. Mentorship is considered a top-down or peer-to-peer process where it can be formal or informal (Keogh, 2015). Mentoring offers benefits for both mentor and mentee including enhancing skills, career growth, and nurturing professional advancement [32]. While it can be done individually, uptake of mentoring can be limited and hence can be enabled by organisational strategies and approaches as noted here. Mentorship was found to be effective at all career levels: early, mid-, and senior levels in academia, clinical care, and global health [27–29, 31]. According to van Dongen et al. [33], the postdoctoral participants of the *Leadership Mentoring in Nursing Research Programme* successfully incorporated new knowledge, abilities, and perspectives into their routine activities. This led to improved leadership techniques, wise career decisions, productive research projects, and international collaborations in research, academia, clinical practice, and management.

Leadership training is also a potentially effective intervention for advancing women into leadership. Here we note when implemented at an organisational level, targeting women nurses in healthcare leadership can enable leadership advancement. Tsoh et al. [30] have identified six core leadership competencies that are potentially effective for leadership training in order to have an individual, interpersonal, and institutional-level impact. These core competencies are “self-awareness, critical thinking, effective communication, inclusion, collaboration, and empowered professionalism” (p. 2). This is consistent with broader literature [1, 20]. Leadership training alone is ineffective without providing leadership opportunities to apply learned knowledge and skills [1]. Hence, the context of organisational change to ensure both training and opportunity is key.

The nursing profession, gender, and other intersectional issues are unique challenges to leadership advancement in nursing [1]. Here we report on current barriers to women nurses' advancement in healthcare leadership. The nursing workforce is primarily constituted of women, and how women are treated in a culture typically reflects how nurses are regarded there [9]. These included a lack of confidence and assertiveness, a dearth of leadership training, fewer opportunities for mentorship and networking, limited career pathways, and a poor understanding of role expectations [1, 27, 28, 31]. Mentoring and leadership development can be utilised as tools to promote self-awareness and engagement. In addition, unconscious gender bias, the glass ceiling for women nurses' career advancement, and power imbalances were noted [1, 28, 31] and were consistent with broader literature [1, 21]. The chance of failure increases when women are given opportunities to develop their ability without being supported in meeting their capacity needs to take advantage of those possibilities [18].

### 4.1. Limitations

The focus of this systematic review included organisational strategies specifically within nursing. It did not capture interventions targeting individuals. Further research is needed to investigate nursing academia career pathways and early career nursing insights into barriers, facilitators, and tailored interventions, alongside implementation strategies to implement and scale effective interventions at the organisational level.

The included studies were observational studies. The content of the mentorship and training has not been reported in detail. Future research is needed to further investigate intervention characteristics.

## 5. Conclusion

In this systematic review, organisational-level strategies for advancing women nurses in healthcare leadership have been captured. Mentorship, leadership training, career planning and career coaching, sponsorships, and networking opportunities were found to be effective organisational-level strategies regardless of nurses' career stage. The necessity of focusing on strategies and interventions at the organisational level was also emphasised to improve women nurses in healthcare leadership, as was capturing but moving beyond the barriers to enablers and implementation. This work contributes to a competitively funded project by the NHMRC, which aims to facilitate systems and organisational change to advance women into healthcare leadership.

## Figures and Tables

**Table 1:** Core themes identified from qualitative and mix-methods studies investigating organisational strategies for women nurses' leadership development.

Organisational strategies	[26]	[27]	[28]	[29]	[30]	[31]
Mentorship		✓	✓	✓		✓
Leadership development programs/training		✓	✓	✓	✓	
Leadership opportunities		✓	✓	✓		
Sponsorships				✓		
Career planning/career coaching	✓					
Providing networking opportunities						✓
Targeted recruitment to improve women representation in leadership roles			✓			
Orientation to leadership role		✓				

**Table 2:** Organisational strategies and leadership barriers and facilitators by career stage and setting.

Career stage	Study ref	Risk of bias	Organisational strategies	Barriers	Facilitators
Early career nursing academia	[27]	Moderate	(i) Career assistance (ii) Orientation programs (iii) Mentorships (iv) Leadership training; educational interventions such as small group work with the experienced leader/case scenario presentations	*Systemic barriers* (i) Available nursing leadership training and mentoring programs are focusing on clinicians rather than academics *Individual barriers* (i) Not viewed as leaders in a new role (ii) Not being assertive (iii) Lack of confidence and autonomy (iv) Poor understanding of teaching-learning practices, role, and expectations as academic	(i) Providing leadership opportunities

Senior-level nursing academia	[30]	Low	(i) Leadership development program	Not reported	Not reported

Mid-career clinical care	[26]	High	(i) Career planning opportunities aiming at mid-career nurses' personal and professional development (ii) Structured career coaching	Not reported	Not reported
Senior-level clinical care	[31] [29]	Moderate Low	(i) Mentorship (ii) Providing networking opportunities Leadership development programs Mentorship and sponsorships	*Systemic barriers* (i) Glass ceiling for higher leadership roles (ii) Inability to implement change results in frustration (iii) Unable to take issues forward (iv) Lack of clear career pathway/no internal training Not reported	(i) Management training (ii) Formal mentoring training for mentors (iii) Professional graduate degree/leadership training/executive coaching/fellowship training (iv) Mentorship (v) Sponsorship (vi) Networking (vii) Family support (viii) Leadership experience (ix) Career planning (x) Taking risks

Early career Mid-career Senior level in global health	[28]	Low	(i) Incentivising mentorships (ii) Implementing targeted recruitment to improve women representation in leadership roles (iii) Providing funding for travel (iv) Family friendly policies to address work-life balance issues (vi) Blinding recruitment and unconscious gender bias training to eliminate system/culture/gender bias for leadership roles (v) Leadership training	*Systemic barriers* (i) Lack of mentorship/sponsorships (ii) Gender bias for women for leadership (iii) Lack of women mentors *Individual barriers* (i) Lack of confidence/assertiveness (ii) Work-life balance *Low and middle-income countries* *Systemic barriers* (i) Lack of opportunities (ii) Lack of funding for meetings and networking (iii) Workload (iv) Travel requirement (v) Lack of leadership training (vi) Intersection for women of colour for leadership (vii) Normalisation of male leadership styles *Individual barriers* (i) Safety concerns	Not reported

## Supplementary Materials

Supplementary Materials Table S1: overview of the included studies. Table S2: risk of bias assessment at the study level. Figure S1: PRISMA flowchart. Figure S2: organisational strategies for advancing women nurses in healthcare leadership by career stages. Table S3: PRISMA 2020 for Abstract Checklist. Table S4: PRISMA 2020 Checklist.
