# Supplementary material for: Organisational Strategies for Women Nurses to Advance in Healthcare Leadership: A Systematic Review
Source: J Nurs Manag. 2023 Dec 7;2023:2678916. doi: 10.1155/2023/2678916 (PMC11918808; doi:10.1155/2023/2678916)
Supplement: Supplementary Materials — Table S1: overview of the included studies. Table S2: risk of bias assessment at the study level. Figure S1: PRISMA flowchart. Figure S2: organisational strategies for advancing women nurses in healthcare leadership by career stages. Table S3: PRISMA 2020 for Abstract Checklist. Table S4: PRISMA 2020 Checklist. [file 2678916.f1.docx]

**ORGANISATIONAL STRATEGIES FOR WOMEN NURSES TO ADVANCE IN HEALTHCARE LEADERSHIP**

Contents

[Table S1: Overview of the included studies 2](#_Toc152130227)

[Table S2: Risk of bias assessment at the study level 3](#_Toc152130228)

[Figure S1: PRISMA flow chart 3](#_Toc152130229)

[Figure S2: Organisational interventions for advancing women nurses in healthcare leadership by career stages 4](#_Toc152130230)

[Table S3: PRISMA 2020 for Abstracts Checklist 5](#_Toc152130231)

[Table S4: PRISMA 2020 Checklist 6](#_Toc152130232)

# Table S1: Overview of the included studies

| Author & Year | Country | Journal | Sample size | Population | Methodology | Data collection & Analysis |
| --- | --- | --- | --- | --- | --- | --- |
| Donner et al., 2001 | Canada | Canadian journal of nursing leadership | 12 | Mid-career nurses (Clinicians) | Qualitative longitudinal study | Narrative Description |
| Halcomb et al., 2016 | Australia | Journal of nursing management | 23 | Early-career nursing academics | Mixed-method | 360^0^ feedback survey based on Fareys’ (1993) Leader/ Manager framework- Descriptive statistics  Semi-structured interviews- thematic analysis |
| Moyer et al., 2018 | Global | Annals of global health | 405 | Respondents, 96.7% of which were women | Mixed-methods | Survey-KPMG Women’s leadership study tool  22 quantitative questions to identify barriers- Descriptive statistics and multivariate logistic regression analysis to find the factors associated with perceived gender bias and career growth in global health career  4 open ended questions to identify potential solutions- Thematic analysis and triangulate with quantitative data. |
| Sexton et al., 2014 | USA | Journal of healthcare management | 20 | Women hospital CEOs | Qualitative | Case study- Narrative Description |
| Tsoh et al. 2019 | USA | Medical education online | 72 | UCSF graduates | Mixed-method longitudinal | Survey- descriptive statistics  Open-ended questions in survey-Thematic analysis |
| Woolnough et al., 2006 | UK | Health service Management Research | 24 | Mental health nurses | Qualitative | Semi-structured telephone interviews  Thematic content analysis |

# Table S2: Risk of bias assessment at the study level

| **Criteria** | **Focus/ criteria** | | **Sample Size** | **Recruitment** | **Baseline Characteristics** | **Confounders considered** | **Interventions** | **Measures** | **Outcomes** | **Analysis** | **Replication** | **Total** |
| --- | --- | --- | --- | --- | --- | --- | --- | --- | --- | --- | --- | --- |
| **High quality / Low risk of Bias** | | | | | | | | | | | | |
| **Tsoh, et al.,** | | 2 | 2 | 2 | 2 | 0 | 2 | 2 | 2 | 2 | 2 | 18 |
| **Moyer, et al.,** | | 2 | 2 | 1 | 2 | 1 | 1 | 2 | 2 | 2 | 1 | 16 |
| **Sexton et al.,** | | 2 | 1 | 2 | 2 | 1 | 2 | 1 | 2 | 2 | 1 | 16 |
| **Moderate quality (8-14)/ Moderate Risk of Bias** | | | | | | | | | | | | |
| **Halcomb et al.,** | | 2 | 1 | 1 | 2 | 1 | 2 | 1 | 1 | 2 | 1 | 14 |
| **Woolnough, et al.,** | | 2 | 1 | 1 | 2 | 1 | 2 | 1 | 1 | 1 | 1 | 13 |
| **Low quality (<7) / High risk of Bias** | | | | | | | | | | | | |
| **Donner,M., and Wheeler, G.** | | 1 | 1 | 1 | 0 | 0 | 0 | 1 | 0 | 1 | 0 | 5 |

# Figure S1: PRISMA flow chart


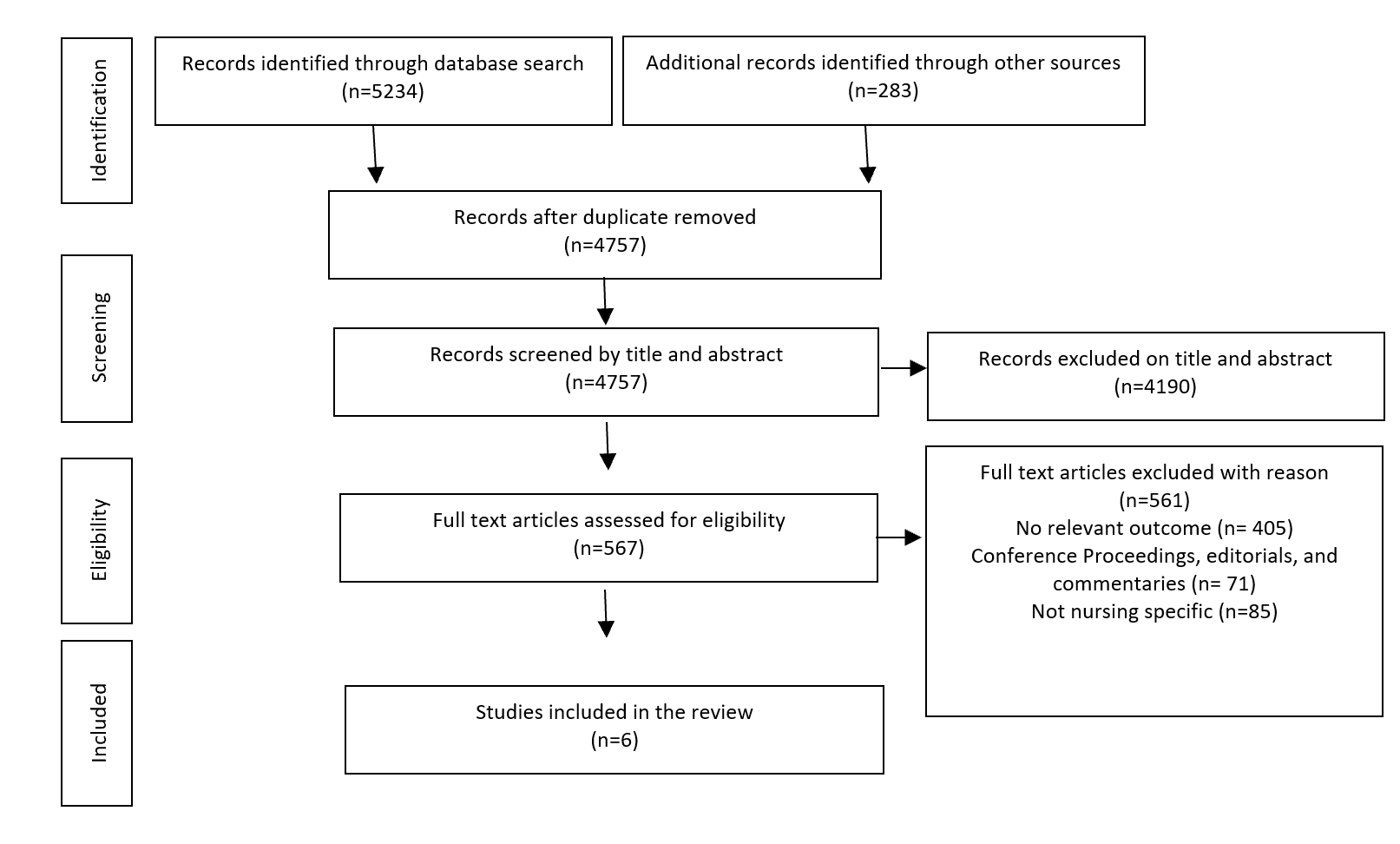


# Figure S2: Organisational interventions for advancing women nurses in healthcare leadership by career stages


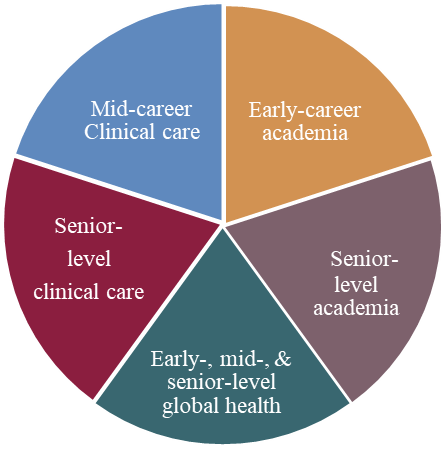


**Organisational**

**Interventions**

- Career assistance
- Orientation
- Mentorships
- Leadership training
- Leadership development programmes
- Mentorship
- Providing networking opportunities
- Leadership training
- Sponsorships
- Career planning opportunities aiming Mid-career nurses’ personal and professional development
- Structured career coaching
- Incentivising mentorships
- Implement targeted recruitment to improve women representation in leadership roles
- Providing funding for travel
- Family-friendly policies to address work-life balance issues
- Blinding recruitment and unconscious
- gender-bias training to eliminate system/
- culture/ gender bias for leadership roles
- Leadership trainings

| **Section and Topic** | **Item #** | **Checklist item** | **Reported (Yes/No)** |
| --- | --- | --- | --- |
| **TITLE** | | |  |
| Title | 1 | Identify the report as a systematic review. | Yes |
| **BACKGROUND** | | |  |
| Objectives | 2 | Provide an explicit statement of the main objective(s) or question(s) the review addresses. | Yes |
| **METHODS** | | |  |
| Eligibility criteria | 3 | Specify the inclusion and exclusion criteria for the review. | Yes |
| Information sources | 4 | Specify the information sources (e.g. databases, registers) used to identify studies and the date when each was last searched. | Yes |
| Risk of bias | 5 | Specify the methods used to assess risk of bias in the included studies. | Yes |
| Synthesis of results | 6 | Specify the methods used to present and synthesise results. | Yes |
| **RESULTS** | | |  |
| Included studies | 7 | Give the total number of included studies and participants and summarise relevant characteristics of studies. | Yes |
| Synthesis of results | 8 | Present results for main outcomes, preferably indicating the number of included studies and participants for each. If meta-analysis was done, report the summary estimate and confidence/credible interval. If comparing groups, indicate the direction of the effect (i.e. which group is favoured). | No  Moved to method section as per Reviewer 1 comment |
| **DISCUSSION** | | |  |
| Limitations of evidence | 9 | Provide a brief summary of the limitations of the evidence included in the review (e.g. study risk of bias, inconsistency and imprecision). | No, in the main text |
| Interpretation | 10 | Provide a general interpretation of the results and important implications. | Yes |
| **OTHER** | | |  |
| Funding | 11 | Specify the primary source of funding for the review. | No, in the main text |
| Registration | 12 | Provide the register name and registration number. | No, in the main text |

# Table S3: PRISMA 2020 for Abstracts Checklist

*From:*  Page MJ, McKenzie JE, Bossuyt PM, Boutron I, Hoffmann TC, Mulrow CD, et al. The PRISMA 2020 statement: an updated guideline for reporting systematic reviews. BMJ 2021;372:n71. doi: 10.1136/bmj.n71

# Table S4: PRISMA 2020 Checklist

| **Section and Topic** | **Item #** | **Checklist item** | **Location where item is reported** |
| --- | --- | --- | --- |
| **TITLE** | | |  |
| Title | 1 | Identify the report as a systematic review. | Page 1 |
| **ABSTRACT** | | |  |
| Abstract | 2 | See the PRISMA 2020 for Abstracts checklist. | Completed & attached |
| **INTRODUCTION** | | |  |
| Rationale | 3 | Describe the rationale for the review in the context of existing knowledge. | Page 3-6 |
| Objectives | 4 | Provide an explicit statement of the objective(s) or question(s) the review addresses. | Page 6 |
| **METHODS** | | |  |
| Eligibility criteria | 5 | Specify the inclusion and exclusion criteria for the review and how studies were grouped for the syntheses. | Page 6 |
| Information sources | 6 | Specify all databases, registers, websites, organisations, reference lists and other sources searched or consulted to identify studies. Specify the date when each source was last searched or consulted. | Page 6 |
| Search strategy | 7 | Present the full search strategies for all databases, registers and websites, including any filters and limits used. | Page 6 |
| Selection process | 8 | Specify the methods used to decide whether a study met the inclusion criteria of the review, including how many reviewers screened each record and each report retrieved, whether they worked independently, and if applicable, details of automation tools used in the process. | Page 7- 8 |
| Data collection process | 9 | Specify the methods used to collect data from reports, including how many reviewers collected data from each report, whether they worked independently, any processes for obtaining or confirming data from study investigators, and if applicable, details of automation tools used in the process. | Page 7-8  Table S1: Overview of the included studies |
| Data items | 10a | List and define all outcomes for which data were sought. Specify whether all results that were compatible with each outcome domain in each study were sought (e.g. for all measures, time points, analyses), and if not, the methods used to decide which results to collect. | Page 7 |
|  | 10b | List and define all other variables for which data were sought (e.g. participant and intervention characteristics, funding sources). Describe any assumptions made about any missing or unclear information. | Not done due to meta-synthesis |
| Study risk of bias assessment | 11 | Specify the methods used to assess risk of bias in the included studies, including details of the tool(s) used, how many reviewers assessed each study and whether they worked independently, and if applicable, details of automation tools used in the process. | Page 7 Table S2: Risk of bias assessment at the study level |
| Effect measures | 12 | Specify for each outcome the effect measure(s) (e.g. risk ratio, mean difference) used in the synthesis or presentation of results. | Not done due to meta-synthesis |
| Synthesis methods | 13a | Describe the processes used to decide which studies were eligible for each synthesis (e.g. tabulating the study intervention characteristics and comparing against the planned groups for each synthesis (item #5)). | Page 11  Table 1  Table S1: Overview of the included studies |
|  | 13b | Describe any methods required to prepare the data for presentation or synthesis, such as handling of missing summary statistics, or data conversions. | Not done due to meta-synthesis |
|  | 13c | Describe any methods used to tabulate or visually display results of individual studies and syntheses. | Page 11  Table 1: Core these emerging from qualitative and mix-methods studies investigating organisational strategies for women nurses’ leadership development  Table S1: Overview of the included studies |
|  | 13d | Describe any methods used to synthesize results and provide a rationale for the choice(s). If meta-analysis was performed, describe the model(s), method(s) to identify the presence and extent of statistical heterogeneity, and software package(s) used. | Page 7 |
|  | 13e | Describe any methods used to explore possible causes of heterogeneity among study results (e.g. subgroup analysis, meta-regression). | Page 7 |
|  | 13f | Describe any sensitivity analyses conducted to assess robustness of the synthesized results. | Not done due to meta-synthesis |
| Reporting bias assessment | 14 | Describe any methods used to assess risk of bias due to missing results in a synthesis (arising from reporting biases). | Page 7 |
| Certainty assessment | 15 | Describe any methods used to assess certainty (or confidence) in the body of evidence for an outcome. | Not done. |
| **RESULTS** | | |  |
| Study selection | 16a | Describe the results of the search and selection process, from the number of records identified in the search to the number of studies included in the review, ideally using a flow diagram. | Page 8  Figure S1: PRISMA flow chart |
|  | 16b | Cite studies that might appear to meet the inclusion criteria, but which were excluded, and explain why they were excluded. | Page 8  Figure S1: PRISMA flow chart |
| Study characteristics | 17 | Cite each included study and present its characteristics. | Page 8 |
| Risk of bias in studies | 18 | Present assessments of risk of bias for each included study. | Page 14 Table 2. Table S2: Risk of bias assessment at the study level |
| Results of individual studies | 19 | For all outcomes, present, for each study: (a) summary statistics for each group (where appropriate) and (b) an effect estimate and its precision (e.g. confidence/credible interval), ideally using structured tables or plots. | Page 11 Table 1  Page 14 Table 2 |
| Results of syntheses | 20a | For each synthesis, briefly summarise the characteristics and risk of bias among contributing studies. | Page 14 Table 2 |
|  | 20b | Present results of all statistical syntheses conducted. If meta-analysis was done, present for each the summary estimate and its precision (e.g. confidence/credible interval) and measures of statistical heterogeneity. If comparing groups, describe the direction of the effect. | Not done due to meta-synthesis |
|  | 20c | Present results of all investigations of possible causes of heterogeneity among study results. | Not done due to meta-synthesis |
|  | 20d | Present results of all sensitivity analyses conducted to assess the robustness of the synthesized results. | Not done due to meta-synthesis |
| Reporting biases | 21 | Present assessments of risk of bias due to missing results (arising from reporting biases) for each synthesis assessed. | Not done due to meta-synthesis |
| Certainty of evidence | 22 | Present assessments of certainty (or confidence) in the body of evidence for each outcome assessed. | Not done due to meta-synthesis |
| **DISCUSSION** | | |  |
| Discussion | 23a | Provide a general interpretation of the results in the context of other evidence. | Page 16-18 |
|  | 23b | Discuss any limitations of the evidence included in the review. | Page 18 |
|  | 23c | Discuss any limitations of the review processes used. | Page 18 |
|  | 23d | Discuss implications of the results for practice, policy, and future research. | Page 18 |
| **OTHER INFORMATION** | | |  |
| Registration and protocol | 24a | Provide registration information for the review, including register name and registration number, or state that the review was not registered. | Page 6- a reference to the overarching systematic review has been included. |
|  | 24b | Indicate where the review protocol can be accessed, or state that a protocol was not prepared. | Page 6 |
|  | 24c | Describe and explain any amendments to information provided at registration or in the protocol. | Page 5 |
| Support | 25 | Describe sources of financial or non-financial support for the review, and the role of the funders or sponsors in the review. | Page 19 |
| Competing interests | 26 | Declare any competing interests of review authors. | Page 19 |
| Availability of data, code and other materials | 27 | Report which of the following are publicly available and where they can be found: template data collection forms; data extracted from included studies; data used for all analyses; analytic code; any other materials used in the review. | Page 19 |

From: Page MJ, McKenzie JE, Bossuyt PM, Boutron I, Hoffmann TC, Mulrow CD, et al. The PRISMA 2020 statement: an updated guideline for reporting systematic reviews. BMJ 2021;372:n71. doi: 10.1136/bmj.n71

For more information, visit: http://www.prisma-statement.org/
